# 

**Published:** 1890-09

**Authors:** 


### CONTENTS FOR SEPTEMBER, 1890.

ORIGINAL COMMUNICATIONS.

Atelectasis Pulmonum. By Irving M. Snow, M. D. .... 65

CLINICAL REPORTS.

On a Temporary Substitute for Milk in the Feeding of Infants. By DeLancey Rochester, M. D. .... 75

SOCIETY PROCEEDINGS.

Philadelphia County Medical Society .... 78
Buffalo Medical and Surgical Association .... 85

PROGRESS IN MEDICAL SCIENCE.

Rhinology, Laryngology and Otology. By Frank Hamilton Potter, M. D. .... 93
Diseases of Children. By DeLancey Rochester, M. D. .... 96
Therapeutic Notes .... 99

CORRESPONDENCE.

Letter from Paris .... 103

EDITORIAL.

Hay Fever .... 107
The Abuse of Hypnotism .... 109
A Mild Protest .... 110
*The Dixie Doctor*—Dr. William Lomax .... 112
Society Meetings .... 112
Medical College Notes .... 113

BOOK REVIEWS.

Diseases of the Rectum and Anus. Their Pathology, Diagnosis and Treatment .... 113
Rheumatism and Gout .... 114
Health Notes for Students .... 115
Annual of the Universal Medical Sciences .... 116
Stories of a Country Doctor .... 117
Leçons de Gynecologie Opératoire .... 117
May's Diseases of Women .... 118
Electricity in the Diseases of Women .... 118
Terminologia Medica Polyglotta .... 119
How to Examine for Life Insurance .... 119
Books and Pamphlets Received .... 120

MISCELLANY.

Recent Patents in Medicine and Surgery .... 124
Literary and Miscellaneous .... 127, 128

